# Vitamin D receptor rs3782905 and vitamin D binding protein rs7041 polymorphisms are associated with hepatocellular carcinoma susceptibility in cirrhotic HCV patients

**DOI:** 10.1186/s12920-023-01749-8

**Published:** 2023-12-08

**Authors:** Asmaa Samir El-masry, Amina M. Medhat, Mahmoud El-Bendary, Rania Hassan Mohamed

**Affiliations:** 1 Department of Biochemistry, Faculty of Science, Ain Shams University, Cairo, Egypt; 2 Department of Tropical Medicine and Hepatology, Faculty of Medicine, Mansoura University, Dakahlia, Egypt

**Keywords:** HCC, HCV, Vitamin D Receptor, Vitamin D Binding Protein, Single nucleotide Polymorphism

## Abstract

**Background:**

The severity of chronic hepatitis C and susceptibility to hepatocellular carcinoma (HCC) are associated with genetic variations within vitamin D receptor (VDR) in several populations. This study aims to determine the significance of the VDRs (rs2228570, rs3782905, rs11568820) and DBP (rs7041) for the susceptibility to HCC in Egyptian patients with chronic HCV infection and their effect on the progression of liver cirrhosis to carcinogenesis.

**Methods:**

Single nucleotide polymorphisms (SNPs) VDR (rs2228570, rs3782905), and DBP rs7041 were genotyped using restriction fragment length-PCR (RFLP-PCR) technique and VDR rs11568820 was genotyped using single strand polymorphism PCR (SSP PCR). These SNPs genotypes, haplotypes and linkage disequilibrium analyses were examined in 299 Egyptian individuals (100 HCV-cirrhotic patients, 99 HCC- HCV patients, and 100 healthy controls).

**Result:**

The VDR rs2228570 CC genotype, VDR rs3782905 GC and CC genotypes, and DBP rs7041 GG genotype are significantly higher in HCC. It is noteworthy that, VDR rs3782905 CC and DBP rs7041 TG genotypes are higher in HCV induced liver cirrhosis than with HCC progression in HCV infected patients. Furthermore, among patients, the relationship between these SNPs and smoking status, gender, and HCC susceptibility was reported.

**Conclusion:**

Among the four investigated SNPs, there are associations between VDR rs3782905 and DBP rs7041 and the HCC progression in Egyptian patients chronically infected with HCV. These SNPs are considered as risk factors in HCV induced liver cirrhosis and HCC. The combinations of these SNPs with smoking status and gender are statistically linked to a high risk of HCC. Future research with a larger sample size of subjects with HCV infection is advised, because chronic liver disease induced by HCV infection is the primary cause of HCC in Egypt. We recommend screening of these SNPs for prediction of LC and HCC development in HCV infected patients, which may improve the used therapeutic protocol. These results suggest that VDR polymorphisms may be potential determinants for HCC susceptibility in Egyptian HCV patients.

**Supplementary Information:**

The online version contains supplementary material available at 10.1186/s12920-023-01749-8.

## Background

Liver cancers accounted for a third of all related deaths and a sixth of all new cancer cases, with an average of 830,180 deaths and 905,677 new cases. Hepatocellular carcinoma (HCC), which makes up 75 to 85%of liver cancer cases, is the most frequent type [1, 2]. Health authorities in Egypt have reported that HCC is considered the most challenging health problem. Over ten years, the number of HCC patients increased by more than twice ([3]. HCC is a complicated, multistage, and multifaceted process [4, 5]). Hepatitis C virus (HCV) infection, a prominent cause of cirrhosis (93%) worldwide [6], is also a risk factor for HCC [7]. There are several known risk factors for HCC, including tobacco use, certain environmental carcinogens, and familial or genetic factors [8].

Vitamin D, which is working through binding with VDR, is involved in cancer and inflammatory liver disease development [9]. Vitamin D receptors (VDR), both nuclear and cytosolic, are required for vitamin D action in cells. VDR ligands affect the expression of 500–1000 genes [10]. Multiple allelic variations exist in the VDR gene located on chromosome 12q. Numerous single nucleotide polymorphisms (SNPs) are studied, some of which are involved in the growth of tumors and chronic liver diseases [11]. Fok1, Bsm1, APa1, and Taq1 are the four SNPs that have been studied the most [12].

The vitamin D-binding protein (DBP) is the most crucial transport protein for vitamin D in the blood. The amount of active vitamin D in the blood greatly influances the DBP levels [13]. To maintain the physiological level of vitamin D, renal tubular cells must produce, filter, and then reabsorb the DBP/25-hydroxyvitamin D [25(OH) D] via VDRs [14]. The highly polymorphic "group-specific component" (GC) gene, which encodes the DBP protein located on chromosome 4 (4q11–13) [15]. The two DBP most common polymorphisms, rs7041 (c.1296 T > G) and rs4588 (c.1307C > A), were described as non-synonymous coding SNP [16].

The involvement of VDR SNPs in HCC development was reported in several populations [17–23] with fluctuating data, but a few VDR SNPs have been examined concerning HCC risk factors in the Egyptian population [24–27], however, it is still unclear in Egyptian patients whether VDR and DBP SNPs could be risk factors in chronic HCV-infected HCC patients. As a result, this study aims to determine the significance of the VDRs (rs2228570, rs3782905, rs11568820) and DBP (rs7041) for the susceptibility to HCC in Egyptian patients with chronic HCV infection and their effect on the progression of liver cirrhosis to carcinogenesis.

## Patients and methods

### Study participants

Prospectively, the 299 Egyptians were chosen from the inpatient and outpatient clinics of the Tropical Medicine Department of the Mansoura University Hospital in Egypt. The participants were classified into 3 groups: 99 HCV-HCC patients, 100 HCV-cirrhotic patients and 100 individuals as controls with no prior history of liver disease (HBV and HCV Negative). Patients with HCV were diagnosed or had been undergoing follow-up care. The HCV infection was confirmed by anti-HCV antibodies and HCV RNA by using ELISA and Real-time PCR tests. HCC patients were diagnosed by serum alpha-fetoprotein (AFP) in addition to imaging by abdominal US and spiral CT. Diagnostic imaging standards with high specificity for HCC ≥ 10 mm have been established by the American College of Radiology through its Liver Imaging Reporting and Data System (LIRADS) and previous AASLD guidelines [28]. These include hyperenhancement in the arterial phase combined with washout appearance and/or capsule appearance. Lesions that did not meet these guidelines or were smaller than 1 cm were not incorporated into this study. Liver cirrhosis (LC) in patients was diagnosed based on combined clinical, laboratory, and radiological findings. In some unclear cases, certain imaging tests, including transient or magnetic resonance elastography (MRE), CT and ultrasound may be recommended. Those who had HIV or HBV and autoimmune disease were not included in this study. In accordance with the Declaration of Helsinki and with participants' signed informed permission, the local institutional review board of the Faculty of Medicine at Mansoura University accepted our present patient study.

### Laboratory analyses

A sample of 5 ml of peripheral blood was taken from each participant. Three ml of the sample were collected in dry tubes to obtain sera for evaluation of biochemical parameters. For molecular analysis, 2 ml of the sample were collected in sterile tubes containing ethylene-diamine–tetraacetic acid (EDTA). Full clinical examination and full history were performed on all patients. Baseline measurements of the participants' liver biochemical profile and AFP were made according to the manufacturer's recommendations for each kit.

### Molecular analysis

#### DNA isolation

Genomic DNA was extracted using the the Wizard Genomic DNA Integrity Kit according to the manufacturer's instructions (Promega, Madison, USA). The 1% agarose gel was used to demonstrate the DNA's integrity. Using NanoDrop™ 2000/2000c, the purity and concentration of DNA were assessed (Thermo Fisher Scientific, Waltham, MA, USA). In order to perform SNP genotyping, the extracted DNA is kept at -20 ºC.

#### Genotyping of VDR and DBP

The polymerase chain reaction-restriction fragment length polymorphism (PCR–RFLP) was used to identify the SNPs of rs2228570, rs3782905, and rs7041 Table 1. PCR was performed in a total volume of 12.5 μl using Biometra II thermal cycler (Göttingen, Germany). The PCR cycles were as follows: 94˚C for 7 min, 30 cycles of denaturation at 95 ˚C for 45 s, annealing at the indicated temperatures in Table 1 for 45 s, extension at 72 ˚C for 45 s, and a final extension at 72 ˚C for 5 min.

**Table 1:** The sequence of the primers and PCR–RFLP products characteristics

SNPS	Primer Sequance	Annealing Temprature	Restriction enzyme	Product Size
rs2228570	F: 5'-AGCTGGCCCTGGCACTGACTCTGGCTCT-3'	**C˚60**	BseG I	**CC: 267 bp**
	R: 5'-ATGGAAACACCTTGCTTCTTCTCCCTC-3'			**CT: 267 + 204 + 63 bp**
				**TT: 204 + 63 bp**
rs3782905	F: 5'-AAGACATGGTGTCTGCTTCA-3'	**56.0˚C**	HpyF3 I	**CC: 223 + 81 bp**
				**CG: 304 + 223 + 81 bp**
	R: 5'-GGTTAGATCGATATGTTTGA-3'			**GG: 304 bp**
rs7041	F: 5'-AAATAATGAGCAAATGAAAGAAGAC-3'	**56.0˚C**	BsuR I	**TT: 482 bp**
				**TG: 482 + 298 + 184 bp**
	R: 5'-CAATAACAGCAAAGAAATGAGTAGA-3'			**GG: 298 + 184 bp**
rs11568820	F1: 5'-AGGATAGAGAAAATAATAGAAAACATT-3'	**45.0˚C**		**GG: 297 + 110 bp**
	R1: 5'-AACCCATAATAAGAAATAAGTTTTTAC-3'			**GA: 297 + 235 + 110 bp**
	F2: 5'-TCCTGAGTAAACTAGGTCACAA-3'			**AA: 297 + 235 bp**
	R2: 5'-ACGTTAAGTTCAGAAAGATTAATTC-3'			

Enzyme (BseG I) investigates SNP (rs2228570), Enzyme (HpyF3 I) investigates SNP (rs3782905), Enzyme( BsuR I) investigates SNP (rs7041)

The PCR products were visualized under UV on 2% agarose gel, which was stained with ethidium bromide. After amplification, the PCR products were digested for 1 h and then visualized on a 2.5% agarose gel, as described in Table 1. SNP rs11568820 was genotyped by the single-stranded polymorphism polymerase chain reaction (SSP-PCR), was performed with the following cycling program: 94˚C for 7 min, 30 cycles of denaturation at 95 ˚C for 45 s, annealing at 45^o^ C for 45 s, and extension at 72 ˚C for 45 s, and a final extension at 72 ˚C for 5 min. Annealing temperatures, restriction enzymes, and product size in genotype analysis are summarized in Table 1. The PCR-SSP was performed in a total volume of 12.5 μl reaction. PCR cycles were performed as before in Table 1.

### Statistical analysis

Data were computed and statistically analyzed using the SPSS software program (IBM Corp. Released 2012, Version 21.0. IBM SPSS Statistics for Windows; Armonk, NY, USA). In terms of continous variables, the median was expressed along with the 25^th^ and 75^th^ percntiles.The non parametric Kruskal–Wallis test and Mann–Whitney U tests were employed to look at variations in demographic traits and laboratory databetween groups. Different genotypes for each SNP were defined in number and minor allele frequencies were calculated. Hardy–Weinberg equilibrium (HWE) was assessed for each SNP to insure that the selected SNPs were informative enough for genetic statistical analysis and the selected groups represent our population (HWE; *p-*value > 0.05). The risk was indicated by an odds ratio (OR) and a 95% confidence interval (95% CI). Nominal logistic regression models were used to evaluate the association between the independent studied groups and a nominal dependent genotype model. We also used Chi-square (χ^2^) test to compare qualitative variables in each group separately to check for variations in allele and genotype distribution. Using population genotype data, the SHEsis software (http://analysis.bio-x.cn/myAnalysis.php) performed linkage disequilibrium (LD) construction and haplotype evaluation [29]. All results were two-tailed, and statistical significance was set at *p* < 0.05. The Akaike Information Criterion (AIC) and Bayesian Information Criterion (BIC) were used to determine the best fit model for each SNP. The inheritance model (codominant, dominant, recessive, and overdominant) of these SNPs was constructed using SNP Stats (https://www.snpstats.net/start.htm).

## Results

### Demographic and clinical characteristics

The demographic and clinical characteristics of the studied groups are outlined in Table 2. In our study, we included 39 males and 61 females cirrhotic patients with a median age of 50 (42.25–58.75) and patients with HCC were 46 males and 53 females with a median age of 55 (47–60). The age differences between the three groups were statistically significant (*p* < 0.001), while no significant differencesconcerning gender and smoking were observed.

**Table 2:** Demographic and biochemical characteristics of all studied groups

Parameter	Normal Controls (*N* = 100)	Cirrhotic Patients (*N* = 100)	HCC patients (*N* = 99)	*P* value *N* = 100
**Demographic Data**				
**Age (year)(Median IQR)**	33 (26–45)	50 (42.25–58.75)^a,b^	55 (47–60)^a^	*p* < 0.001
**Gender:** M/F	40/60	39/61	46/53	NS
**Smoking status (n %)**				NS
Non-smokers	56 (56%)	60 (60%)	63 (63.6%)	
Smokers	44 (44%)	40 (40%)	36 (36.4%)	
**Laboratory Data (median IQR)**				
**ALT (IU/L)**	18 (12–23)	82 (68.5–93.5)^a,b^	11 (101–118)^a^	*p* < 0.001
**AST(IU/L)**	19 (14–25)	70 (59–82.25)^a,b^	104 (96–110)^a^	*p* < 0.001
**ALB (g/L)**	3.8 (3.6–4.07)	3.2 (3.1–3.3)^a,b^	2.7 (2.6–2.9)^a^	*p* < 0.001
**Creatinine (mg/dL)**	0.8 (0.70–1.0)	1.6 (1.4–1.7)^a,b^	2.1 (2–2.2)^a^	*p* < 0.001
**T.Billirubin (mg/dL)**	0.56 (0.50–0.70)	1.6 (1.4–1.7)^a,b^	2.6 (2.4–2.8)^a^	*p* < 0.001
**D. Billirubin (mg/dL)**	0.12 (0.07–0.16)	0.69 (0.55–0.79)^a,b^	1.2 (1.1–1.3)^a^	*p* < 0.001
**AFP (ng/mL)**	17 (12.25–20.75)	64.5 (46.25–74.75)^a,b^	715 (495–822)^a^	*p* < 0.001

^a^Significante from normal controls

^b^Significance from HCC patients

As shown in Table 2, the biochemical characteristics ALT, AST,creatinine, T-Bil, D-Bil, and AFP were significantly higher in HCC patients than in healthy controls and HCV cirrhotic patients (*p* < 0.001), while ALB was significantly lower (*p* < 0.001).

### VDR and DBP genotypic distribution

Frequencies of the groups' genotypes and alleles are shown in Table 3. The four SNPs in the groups had genotype distributions that fell within the Hardy–Weinberg equilibrium.

**Table 3:** Association analysis ofgenotype distributions and alleles frequencies of the VDR and DBP SNPs

SNP	Normal Control (*N* = 100)	Cirrhotic patients (*N* = 100)	HCC patients (*N* = 99)	Cirrhotic patients vs normal controls		HCC patients vs normal controls		Cirrhotic patients vs HCC patients	
				**OR (95% CI)**	***P* value**	**OR (95% CI)**	***P* value**	**OR (95% CI)**	***P* value**
**VDR (rs2228570)**									
CC	34 (34%)	54 (54%)	46 (46.5%)	2.279 (1.283–4.033)	*p* < 0.01	1.685 (0.951–2.985)	*p* < 0.05	0.739 (0.423–1.291)	NS
CT	48 (48%)	26 (26%)	32 (32.3%)	0.381 (0.210–0.690)	*p* < 0.001	0.517 (0.291–0.920)	*p* < 0.05	1.359 (0.736–2.512)	NS
TT	18 (18%)	20 (20%)	21 (21.2%)	1.139 (0.561–2.310)	NS	1.122 (0.608–2.474)	NS	1.077 (0.542–2.141)	NS
CTTT	66 (66%)	46 (46%)	53 (53.5%)	0.44 (0.25–0.78)	*p* < 0.001	0.59 (0.33–1.05)	NS	1.35 (0.774–2.361)	NS
C	116 (58%)	134 (67%)	124 (63%)	1.470 (0.978–2.208)	NS	1.121 (0.816–1.814)	NS	0.825 (0.546–1.246)	NS
T	84 (42%)	66 (33%)	74 (37%)	0.630 (0.152–1.021)	NS	0.824(0.551–1.234)	NS	1.211 (0.802–1.829)	NS
**VDR (rs3782905)**									
GG	51 (51%)	19 (19%)	23 (23.2%)	0.225 (0.119–0.425)	*p* < 0.001	0.291 (0.158–0.535)	*p* < 0.001	1.290 (0.651–2.556)	NS
GC	29 (29%)	30 (30%)	45 (45.5%)	1.049 (0.571–1.927)	NS	2.040 (1.136–3.665)	*p* < 0.05	1.944 (1.036–3.482)	*p* < 0.05
CC	20 (20%)	51 (51%)	31 (31.3%)	4.163 (2.223–7.798)	*p* < 0.001	1.82 (0.953–3.488)	*p* < 0.05	0.438 (0.246–0.781)	*p* < 0.01
GCCC	49 (49%)	81 (81%)	76 (67.8%)	0.24 (0.13–0.45)	*p* < 0.001	3.44 (1.87–6.33)	*p* < 0.001	0.77 (0.39–1.5)	NS
G	69 (34%)	132 (66%)	107 (54%)	3.685 (2.438–5.570)	*p* < 0.001	2.232 (1.490–3.341)	*p* < 0.001	0.605 (0.404–0.907)	*p* < 0.05
C	131 (66%)	68 (34%)	91 (46%)	0.271 (0.179–0.410)	*p* < 0.001	0.448 (0.299–0.670)	*p* < 0.001	1.650 (1.104–2.474)	*p* < 0.05
**VDR(rs11568820)**									
GG	0 (0%)	0 (0%)	0 (0%)	–––––––––-	–––	–––––––––-	–––	–––––––––-	–––
GA	100 (100%)	100 (100%)	99 (100%)	–––––––––-	–––	–––––––––-	–––	–––––––––-	–––
AA	0 (0%)	0 (0%)	0 (0%)	–––––––––-	–––	–––––––––-	–––	–––––––––-	–––
G	100 (50%)	100 (50%)	100 (50%)	–––––––––-	–––				
A	100 (50%)	100 (50%)	100 (50%)	–––––––––-	–––				
**DBP (rs7041)**									
TT	61 (61%)	23 (23%)	20 (20.2%)	0.191 (0.103–0.353)	*p* < 0.001	0.162 (0.066–0.305)	*p* < 0.001	0.848 (0.431–1.807)	NS
TG	23 (23%)	49 (49%)	29 (29.3%)	3.21 (1.750–5.913)	*p* < 0.001	1.387 (0.734–2.619)	NS	0.431 (0.240–0.773)	*p* < 0.01
GG	16 (16%)	28 (28%)	50 (50.5%)	2.642 (1.024–4.071)	*p* < 0.05	5.357 (2.757–10.408)	*p* < 0.001	2.624 (1.457–4.724)	*p* < 0.001
TGGG	39 (39%)	77 (77%)	79 (79.8%)	5.24 (2.83–6.69)	*p* < 0.001	6.18 (3.28–11.65)	*p* < 0.001	1.17 (0.59–2.32)	NS
T	145 (72%)	95 (48%)	129 (65%)	0.343 (0.226–0.520)	*p* < 0.001	0.709 (0.463–1.086)	NS	1.082 (0.695–1.684)	NS
G	55 (28%)	105 (52%)	69 (35%)	2.913 (1.921–4.418)	*p* < 0.001	1.410 (0.920–2.159)	NS	1.410 (0.920–2.159)	NS

*P* p-value is significant < 0.05, *NS* Non-significante

#### VDR (FokI rs2228570) genotypes distribution

**Fig. 1:**
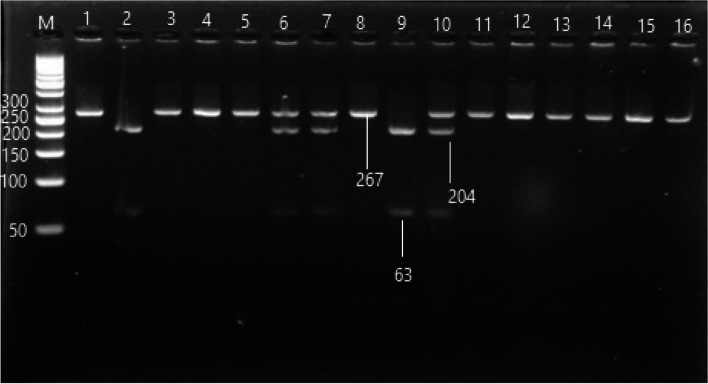
PCR–RFLP product of rs2228570 polymorphism, lane (M) 50 bp ladder, lanes (1,3,4,5,8,11,12,13,14,15,16) were CC genotype 267 bp, Lanes (6,7,10) were CT genotype 267, 204 and 63 bp, and lanes (2,9) were TT genotype 204 and 63 bp (Croped)

The PCR–RFLP targeting rs2228570 was represented in Fig. 1. The genotypic frequency of VDR rs2228570 in HCV cirrhotic and HCC patients had a high prevalence of CC genotype compared to healthy control (OR = 2.279; CI: 1.283–4.033, *p* < 0.01, OR = 1.685; CI: 0.951–2.985, *p* < 0.05), respectively. The distribution of genotypes and alleles were insignificant differences by comparing HCC patients to both the healthy control group and the cirrhotic group.

#### VDR (BsmI rs3782905) genotypes distribution

**Fig. 2:**
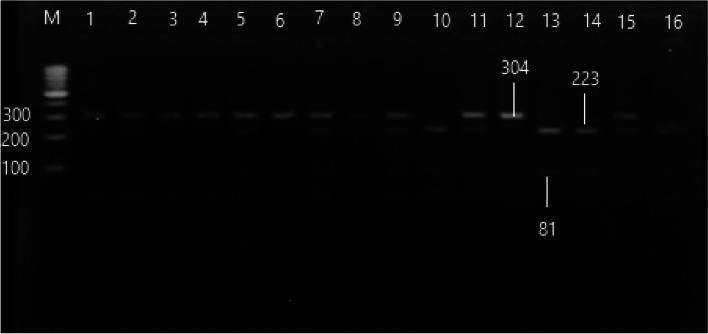
PCR–RFLP product of rs3782905 polymorphism, lane (M) 100 bp ladder, lanes (1,3,4,6,12) were GG genotype 304 bp, lanes (2,5,7,8,9,11,15) were CG genotype 304, 223 and 81 bp, and lanes (10,13,14,16) were CC genotype 223 and 81 bp (cropped)

The PCR–RFLP targeting BsmI rs3782905 was represented in Fig. 2. The genotypic frequency of rs3782905 in HCV cirrhotic patients had a high prevalence of CC genotype than the healthy control (OR = 4.163; CI: 2.223–7.798, *p* < 0.001) with a predominance of the G allele (OR = 3.685; CI: 2.438–5.570, *p* < 0.001). However, HCC patients had a high prevalence of GC and CC genotypes in comparison with healthy control (OR = 2.040; CI: 1.136–3.665, *p* < 0.05, OR = 1.82; CI: 0.953–3.488, *p* < 0.05 respectively), and high GC genotype frequency in comparison with HCV cirrhotic patients (OR = 1.944; CI: 1.036–3.482, *p* < 0.05) with a predominance of the C allele (1.650 (1.104–2.474). The CC genotype is less frequent in HCC than in HCV cirrhotic patients (OR = 0.438; CI: 0.246–0.781, *p* < 0.01).

#### VDR (Cdx-2 rs11568820) genotype distribution

**Fig. 3:**
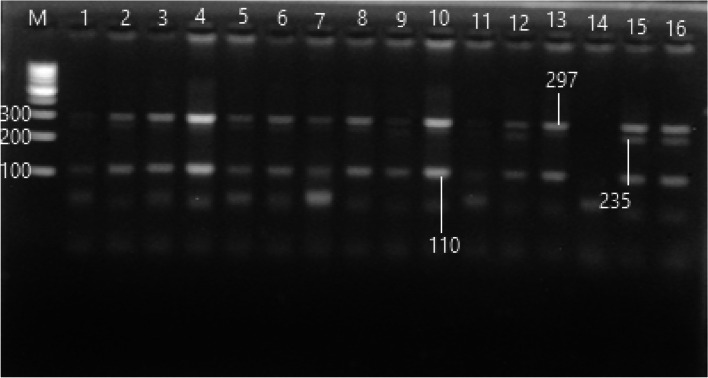
PCR-SSR product of rs11568820 polymorphism, lane (M) 100 bp ladder, lanes (1,16) were GA genotype 297, 235 and 110 bp (croped)

The PCR-SSR targeting rs11568820 was represented in Fig. 3. Regarding rs11568820 polymorphism, we have detected only one genotype in all participants (GA genotype). This genotype and allele frequencies in all studied groups did not differ significantly.

#### DBP( rs7041) genotypes distribution

**Fig. 4:**
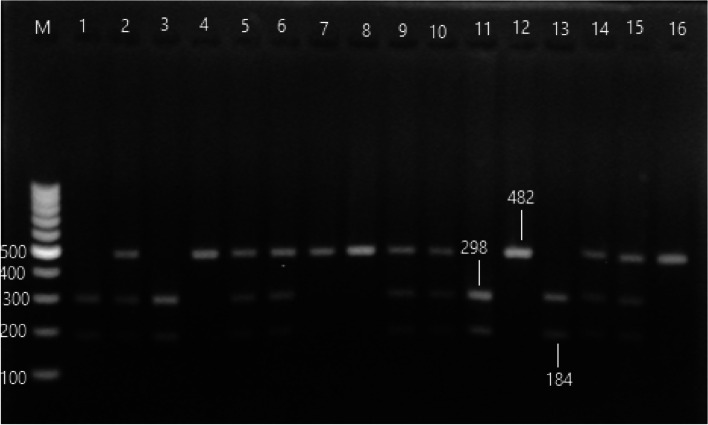
PCR–RFLP product of DBP rs7041 polymorphism, lane (1) 100 bp ladder, lanes (4,7,812,16) were TT genotype 482 bp, lanes (2,5,7,9,10,14,15) were TG genotype 482, 298 and 184 bp, and lanes (1,3,11,13) were GG genotype 298 and 184 bp

The PCR–RFLP targeting DBP, rs7041 was represented in Fig. 4. The genotypic frequency of rs7041 in HCV cirrhotic patients had a high prevalence of TG genotype than the healthy control (OR = 3.21; CI: 1.750–5.913, *p* < 0.01) with significant allelic distributions and predominance of the G allele (OR = 2.913; CI: 1.921–4.418, *p* < 0.01), while HCC patients had a high prevalence of GG genotype compared to both healthy control (OR = 5.357, CI: 2.757–10.408, *p* < 0.001) and cirrhotic patients (OR = 2.624, CI: 1.457–4.724,* p* < 0.001). The TG genotype is less frequent in HCC than in HCV cirrhotic patients (OR = 0.431; CI: 0.240–0.773, *p* < 0.01).

### Haplotype analysis

Estimating haplotypes of rs2228570, rs3782905, rs11568820, and rs7041 polymorphisms in all studied groups gave eight haplotypes as shown in Table 4. HCC patients had a high prevalence of CGG haplotype than the healthy control group and HCV cirrhotic group (OR = 2.85; CI: 1.18–6.89, *p* < 0.05, OR = 2.16; CI: 1.02–4.55, *p* < 0.05, respectively) and TTG haplotype when comparing with HCV cirrhotic group (OR = 3.60 CI: 1.39–9.28, *p* < 0.001). Interestingly, the CGC haplotype was significantly associated with HCV cirrhotic and HCC patients compared with the healthy control group (OR = 8.90 CI: 2.98–26.527, *p* < 0.001, OR = 5.34 CI: 2.08–13.74, *p* < 0.001, respectively). Also, the TGC haplotype showed a significant elevation in HCC patients compared with the healthy control group (OR = 14.74, CI: 8.38–64.26, *p* < 0.001).The absence of linkage disequilibrium (LD) indicated that the studied SNPs rs2228570, rs3782905 and rs7041 loci are not linked to be inherited together between 3 studied SNPs (D = 0.046, r2 = 0.040), rs3782905 andrs7041 (D = 0.035, r2 = 0.0336), rs2228570 and rs3782905 (D = 0.0312, r2 = 0.0225). We have also analysed the model of inheritance and found the best fit ineritance model with the lowest AIC for VDR (rs2228570), DBP (rs7041), VDR (rs3782905) between the different models (codominant, dominant, recessive, and overdominant) as shown in Supplementary Table) 1S and 2S.

**Table 4:** Comparison of all groups under study's VDR and DBP gene polymorphisms' association haplotypes

Haplotypes	Normal Control (*N* = 100)	Cirrhotic patients (*N* = 100)	HCC patients (*N* = 99)	Cirrhotic patients vs normal controls		HCC patients vs normal controls		HCC patients vs Cirrhotic patients	
				**OR (95% CI)**	***P* value**	**OR (95% CI)**	***P* Value**	**OR (95% CI)**	***P* Value**
CTG	24.89%	9.74%	10.2%	0.312 (0.136–0.731)	*p* < 0.01	0.351 (0.157–0.780)	*p* < 0.01	1.123 (0.435–2.896)	NS
CTC	17.4%	19.39%	14.4%	1.145 (0.555–2.36)	NS	2.02 (0.88–4.62)	NS	1.06 (0.51–2.21)	NS
CGG	11.54%	10.37%	19.63%	0.898 (0.363–2.223)	NS	2.85 (1.18–6.89)	*p* < 0.05	2.16 (1.02–4.55)	*p* < 0.05
TTG	20.06%	5.35%	5.37%	0.210 (0.075–0.585)	*p* < 0.01	0.73 (0.25–2.15)	NS	3.60 (1.39–9.28)	*p* < 0.001
CGC	4.18%	27.49%	18.33%	8.9 (2.98–26.527)	*p* < 0.001	5.34 (2.08–13.74)	*p* < 0.001	1.59 (0.69–3.67)	NS
TGC	2.77%	6.1%	16.43%	3.129 (0.615–15.89)	NS	14.74 (3.38–64.26)	*p* < 0.001	1.57 (0.71–3.45)	NS
TGG	9.0%	8.54%	10.77%	0.879 (0.324–2.385)	NS	1.48 (0.60–3.64)	NS	0.51 (0.18–1.43)	NS
TTC	10.16%	13.02%	4.81%	1.345 (0.560–3.230)	NS	0.52 (0.13–2.16)	NS	1.33 (0.40–4.41)	NS

*p* *p*-value is significant < 0.05, *NS* Non-significante

### The correlation of SNPs to smoking and gender

In non-smoker patients, the genotypic frequency of rs7041 had a high prevalence of the GG genotype (OR = 2.23; CI: 1.07–4.64, *p* < 0.05). However in smoker patients, the genotypic frequency of rs7041 had a high prevalence of GG genotype (OR = 3.44; CI: 1.28–9.26, *p* < 0.05). Also, in non –smoker patients the genotypic frequency of DBP rs7041 had a high prevalence of GG genotype (OR = 2.23; CI: 1.07–4.64, *p* < 0.05), as shown in Table 5. Furthermore, in females the genotypic frequency of DBP rs7041 had a high prevalence of GG genotype (OR = 3.94; CI: 1.80–8.61, *p* < 0.01). In males the genotypic frequency VDR rs3782905 had also a high prevalence of GC genotype (OR = 2.56; CI: 1.03–6.30, *p* < 0.05), as shown in Table 6.

**Table 5:** Smoking status and SNPs of VDR and DBP gene association analyses in cirrhotic and HCC patients

	**Nonsmokers**			**Smokers**		
SNPs	**Cirrhosis** ***N* = 60** **(N %)**	**HCC** ***N* = 63** **(N %)**	***P* value** OR(95% CI)	**Cirrhosis** ***N* = 40** **(N %)**	**HCC** ***N* = 36** **(N %)**	***P* value** **OR** (95%CI)
VDR(rs2228570)						
CC	32 (53.3)	29(46)	NS 0.75 (0.37–1.52)	22 (55)	17 (47.2)	NS 0.73 (0.30–1.81)
CT	18 (30)	18(28.6)	NS 0.93 (0.43–2.03)	8 (20)	14 (38.9)	NS 2.55 (0.91–7.09)
TT	10 (16.7)	16 (25.4)	NS 1.70 (0.70–4.12)	10 (25)	5 (13.9)	NS 0.49 (0.15–1.58)
VDR(rs3782905)						
GG	13 (21.7)	14 (22.3)	NS 1.03 (0.44–2.43)	6 (15)	9 (25)	NS 1.88 (0.59–5.97)
GC	18 (30)	29 (46)	NS 1.99 (0.95–4.18)	12 (30)	16 (44.4)	NS 1.87 (0.73–4.79)
CC	29 (48.3)	20 (31.7)	NS 0.50 (0.24–1.04)	22 (55)	11 (30.6)	*p* < 0.05 0.36 (0.14–0.93)
DBP (rs7041)						
TT	16 (26.6)	11 (17.5)	NS 0.59 (0.25–1.38)	7 (17.5)	9 (25)	NS 1.57 (0.52–4.77)
TG	25 (41.7)	20 (31.7)	NS 0.65 (0.31–1.36)	24 (60)	9 (25)	*p* < 0.01 0.22 (0.08–0.59)
GG	19 (31.7)	32 (50.8)	*p* < 0.05 2.23 (1.07–4.64)	9 (22.5)	18 (50)	*p* < 0.05 3.44 (1.28–9.26)

**Table 6:** Gender and SNPs of VDR and DBP geneassociation analyses in cirrhotic and HCC patients

	**Male**			**Female**		
SNPs	**Cirrhosis** ***N* = 39** **(N %)**	**HCC** ***N* = 46** **(N %)**	***P* value** **OR (95% CI)**	**Cirrhosis** ***N* = 61** **(N %)**	**HCC** ***N* = 53** **(N %)**	***P* value** **OR(95% CI)**
VDR(rs2228570)						
CC	22 (56.5)	21 (45.7)	NS 0.65 (0.28–1.53)	32 (52.5)	25 (47.1)	NS 0.81(0.39–1.69)
CT	10 (25.6)	14 (30.4)	NS 1.27 (0.49–3.29)	16 (26.2)	18 (34)	NS 1.45 (0.65–3.24)
TT	7 (17.9)	11 (23.9)	NS 1.44 (0.50–4.16)	13 (21.3)	10 (18.9)	NS 0.86 (0.34–2.16)
VDR(rs3782905)						
GG	7 (17.9)	9 (19.6)	NS 1.11 (0.37–3.33)	12 (19.7)	14 (26.4)	NS 1.47 (0.613.53)
GC	11 (28.3)	23 (50)	*p* < 0.05 2.56 (1.03–6.30)	19 (31.1)	22 (41.5)	NS 1.57 (0.733.39)
CC	21 (53.8)	14 (30.4)	*p* < 0.05 0.38 (0.15–0.91)	30 (49.2)	17 (32.1)	NS 0.49 (0.231.05)
DBP(rs7041)						
TT	13 (33.4)	11 (23.9)	NS 0.63 (0.24–1.63)	10 (16.4)	9 (17)	NS 1.04 (0.39–2.80)
TG	16 (41)	18 (39.1)	NS 0.92 (0.38–2.21)	33 (54.1)	11 (20.8)	*p* < 0.001 0.22 (0.10–0.51)
GG	10 (25.6)	17 (37)	NS 1.70 (0.67–4.33)	18 (29.5)	33 (62.2)	*p* < 0.01 3.94 (1.80–8.61)

## Discussion

In chronic hepatitis C patients, the precise process by which HCC develops, taking into account both host and viral elements, remains uncertain [30]. The high gender disparity in HCC is caused by differences in how each gender is exposed to the causal agents, as well as genetic factors including inflammatory cytokine and growth factor receptor gene polymorphisms [23, 30–32]. Finding more genetic factors that affect the transcription of particular regulatory genes could therefore help to identify high-risk groups. Therefore, the current study's goal was to determine whether there was an association between the HCC in cirrhotic HCV patients in the Egyptian population and SNP of VDR (FOKI rs 2228570, BsmI rs 3782905, Cdx-2 rs 11568820) and DBP(rs7041).

The overall HCC rate was significantly greater in patients aged 55 and older. This finding is the line with Yi et al. [33], who found that male sex and age > 55 in a Chinese population were linked to a higher risk of having HCC. Regarding laboratory data, it was found that the ALT, AST, ALB, creatinine, T-Bil, D-Bil, and AFP levels were significantly different across the three groups. According to Barooah et al. [34] and Raafat et al. [35], they observed higher serum levels of ALT, AST, and bilirubin in HCC patients than in chronic liver disease patients. Additionally, Du et al. [36] observed the risk of HCC in patients with ALT elevation (1.82–2.75-fold). Individuals with chronic HBV who had chronically abnormal ALT may have been included in or referred to by the population that had high ALT. HCC was typically observed in patients with ALT rise, particularly ALT flare (mean 3 months in China). This demonstrates that ALT flare is more likely a predictor than a cause of the development of HCC. Turshudzhyan and Wu [37] also observed an elevation in serum AFP in HCC patients.

Several polymorphisms in the VDR genes, including BsmI, FokI, TaqI, and Cdx-2, have been reported in recent years at various loci. These polymorphisms in the VDR gene can change the activity of VDR proteins [11]. There is a research in Nigerian population confirm that some randomized clinical control trials suggesting vitamin D related mechanism is vital in HCC progression [20]. In the present study, in both cirrhotic and HCC patients, rs2228570 had a significantly high prevalence of CC genotypes. As a result, the CC genotype may be more than a two-fold risk factor for liver cirrhosis, and also for a little rise in the risk of HCC. This is in line with Tsounis et al. [38], who reported that the development of cirrhosis is related to homozygosity for the dominant trait of FokI variants. Contrary to Mohammed et al. [26] findings, HCC risk was considerably higher among those with the FokI (TT) genotype. Also, Moemen et al. [27] reported that the homozygosity for the recessive FokI allele (TT) was associated with increased tumor size and higher AFP levels in Egyptian patients with HCV-related HCC.

In this study, we noticed a high prevalence of the TG genotype for rs7041, suggesting that the TG genotype might be a three-fold risk factor for liver cirrhosis compared to healthy controls with significant allelic distributions. The G allele is correlated with an approximately three-fold increase in liver cirrhosis risk. Nevertheless, the GG genotype may be considered a risk factor for HCC and HCV cirrhotic patients because of the high prevalence in HCC and HCV cirrhotic patients. These results came in agreement with Peng et al. [39], who reported that rs7041 GG and the rs7041 TG/GG genotypes 'were correlated with a significantly increased HCC risk in HBV Chinese patients. Also, similar results were reported in Thailand populations by Maneechay et al. [40], which showed that the risk genotypes (TG/GG) in rs7041 were associated with lung cancer. Also, Chupeerach et al. [41] revealed a correlation between colorectal cancer to the same genotypes.

Data on BsmI polymorphism in liver diseases are scanty. The VDR gene polymorphism at rs3782905 in HCV cirrhotic patients exhibited a significant incidence of CC genotype in this study, suggesting that CC genotype might be a risk factor for LC four times higher than healthy controls. The HCC patients had a high prevalence of GC and CC genotypes that might be considered as a risk factor for HCC in comparison with healthy control. The GC genotype increases the risk for the prognosis of LC to HCC. In Egyptian populations, the GC genotype was correlated to a nearly two-fold increase in HCV-related HCC risk. In contrast to Mohamed et al. [26], it was reported that there was no significant association between rs3782905 polymorphism and HCC development.

It is noteworthy that, VDR rs3782905 CC and DBP rs7041 TG genotypes are more associated with a higher risk of HCV-induced liver cirrhosis than with HCC progression in HCV-infected patients. Furthermore, among patients, the relationship between these SNPs and smoking status, gender, and HCC susceptibility was reported.

Looking at the rs11568820 polymorphism, we detected only one genotype in this SNP in all participants (GA genotype). In line with Peng et al. [39], who studied the association between rs11568820 SNP and HBV-related HCC risk in the Chinese population, we found that no significant association between the frequency of alleles and genotypes of rs11568820 polymorphism within the three studied groups with the development of the HCV-related HCC. In contrast, Dai et al. [42] demonstrated that the rs11568820 polymorphism is believed to contribute to HCC carcinogenesis.

Our results indicated that there is no linkage disequilibrium between the 3 studied SNPs, which indicates that the studied SNPs loci are not linked to be inherited together [43]. The results also suggested that the TGC, CGC, and CGG haplotypes are risk factors for HCC, while the CGG and TTG haplotypes increase the chance of LC turning into HCC.

There is a long list of risk factors for chronic hepatitis patients' condition progressing to HCC. In determining HCC risk, smoking seems to correlate with HBV and HCV [42]. HCC is also known to be more common in men, and numerous SNPs have been linked to a higher genetic susceptibility to the disease [44]. In this study, there was no significant association between VDR SNPs (rs2228570 and rs3782905) and smoking status in HCC risk, which agreed with Galal et al., [24]. However, the GG genotype of DBP (rs7041) SNP was significantly associated with disease progression in both groups (non-smokers and smokers). By looking at gender, in males, the GC genotype of the rs3782905 SNP might be a risk factor for HCC. On the other hand, the GG genotype of rs7041 SNP was significantly associated with increasing LC to HCC in females. Our results were in the same line with Chuang et al. [44] and Yang et al. [45].

## Conclusion

The current study provided evidence that among the four studied SNPs, rs3782905 and rs7041 might be viewed as risk factors for either the progression of chronic HCV liver disease into HCC in Egyptian patients. Up to our knowledge, this is the first study to include the genetic variant of DBP in association to the HCV infected LC and HCC Egyptian patients. In addition, we highlighted the role of VDR and DBP SNPs with smoking status as well as gender in HCC susceptibility. We recommend screening of these SNPs for prediction of LC and HCC development in HCV infected patients, which may improve the used therapeutic protocol. These results suggest that VDR polymorphisms may be potential determinants for HCC susceptibility in Egyptian HCV patients. Future research with larger sample size of subjects with HCV infection is advised, because chronic liver disease induced by HCV infection is the primary cause of HCC in Egypt.

### Supplementary Information

**Additional file 1: Table 1S.** Different inheritance models analysis of the SNPs between Cirrhosis and control groups.** Table 2S.** Different inheritance models analysis of the SNPs between HCC and control groups.

## Data Availability

The datasets used and/or analysed during the current study are available from the corresponding author on reasonable request.

## References

[1] Zhang H, Zhang W, Jiang L, Chen Y (2022). Recent advances in systemic therapy for hepatocellular carcinoma. Biomark Res.

[2] Bray F, Ferlay J, Soerjomataram I, Siegel RL, Torre LA, Jemal A (2018). Global cancer statistics 2018: GLOBOCAN estimates of incidence and mortality worldwide for 36 cancers in 185 countries. CA Cancer J Clin.

[3] Rashed WM, Kandeil MAM, Mahmoud MO, Ezzat S (2020). Hepatocellular Carcinoma (HCC) in Egypt: A comprehensive overview. J Egypt Natl Canc Inst.

[4] Wei J, Fang D (2021). Endoplasmic reticulum stress signaling and the pathogenesis of hepatocarcinoma. Int J Mol Sci.

[5] Farid K, Elalfy H, Abo El-Khair SM, Elgamal H, Besheer T, Elmokadem A (2020). Prognostic value of vascular endothelial growth factor in both conventional and drug-eluting beads transarterial chemoembolization for treatment of unresectable hepatocellular carcinoma in HCV patients. Expert Rev Gastroenterol Hepatol.

[6] Yang JD, Kim WR, Coelho R, Mettler TA, Benson JT, Sanderson SO (2011). Cirrhosis is present in most patients with hepatitis B and hepatocellular carcinoma. Clin Gastroenterol Hepatol.

[7] Jindal A, Thadi A, Shailubhai K (2019). Hepatocellular carcinoma: etiology and current and future drugs. J Clin Exp Hepatol.

[8] Yang JD, Hainaut P, Gores GJ, Amadou A, Plymoth A, Roberts LR (2019). A global view of hepatocellular carcinoma: trends, risk, prevention, and management. Nat Rev Gastroenterol Hepatol.

[9] Kamen DL, Tangpricha V (2010). Vitamin D and molecular actions on the immune system: modulation of innate and autoimmunity. J Mol Med (Berl).

[10] Haussler MR, Haussler CA, Whitfield GK, Hsieh JC, Thompson PD, Barthel TK (2010). The nuclear vitamin D receptor controls the expression of genes encoding factors which feed the "Fountain of Youth" to mediate healthful aging. J Steroid Biochem Mol Biol.

[11] Abudeif A, Galal G, Mohammad A, Agamy M, Ahmad N, Fahmy N (2019). VDR gene polymorphisms and risk of hepatocellular carcinoma. Sohag Med J.

[12] Selvaraj P, Chandra G, Jawahar MS, Rani MV, Rajeshwari DN, Narayanan PR (2004). Regulatory role of vitamin D receptor gene variants of Bsm I, Apa I, Taq I, and Fok I polymorphisms on macrophage phagocytosis and lymphoproliferative response to mycobacterium tuberculosis antigen in pulmonary tuberculosis. J Clin Immunol.

[13] Abdella NA, Mojiminiyi OA (2018). Vitamin D-binding protein clearance ratio is significantly associated with glycemic status and diabetes complications in a predominantly vitamin D-deficient population. J Diabetes Res.

[14] Thrailkill KM, Jo CH, Cockrell GE, Moreau CS, Fowlkes JL (2011). Enhanced excretion of vitamin D binding protein in type 1 diabetes: a role in vitamin D deficiency?. J Clin Endocrinol Metab.

[15] Speeckaert M, Huang G, Delanghe JR, Taes YE (2006). Biological and clinical aspects of the vitamin D binding protein (Gc-globulin) and its polymorphism. Clin Chim Acta.

[16] Jorde R, Schirmer H, Wilsgaard T, Mathiesen EB, Njølstad I, Løchen ML (2015). The DBP phenotype Gc-1f/Gc-1f is associated with reduced risk of cancer. The Tromsø study. PLos One.

[17] Jiawei R, Xukun Wu, Xiaozhuan Z, Ronghai D and YiMa. Vitamin D Receptor FokI Polymorphism and Risk of Hepatocellular Carcinoma in HBV-Infected Patients. Hepta Mon. 2018. 10.5812/heptamon.85075.

[18] Brait B, Silva SB, Aguiar FL, Ferreira R, Brancat C, Brancati O (2022). Genetic polymorphisms related to the vitamin D pathway in patients with cirrhosis with or without hepatocellular carcinoma (HCC). Ecancer Medicalscience.

[19] Tourkochristou E, Mouzaki A, Triantos C (2023). Gene polymorphisms and biological effects of vitamin D receptor on nonalcoholic fatty liver disease development and progression. Mol Sci.

[20] Adelani I, Rotimi O, Maduagwu E, Rotimi S (2021). Vitamin D: possible therapeutic roles in hepatocellular carcinoma. Frontiser.

[21] Hoan N, Khuyen N, Giang D, Binh M, Toan N, Anh D (2019). Vitamin D receptor ApaI polymorphism associated with progression of liver disease in Vietnamese patients chronically infected with hepatitis B virus. BMC Med Genet.

[22] Barooh P, Saikia S, Bharadwaj R, Sarmah P, Bhattacha M, Goswami B (2019). Role of VDR, GC, and CYP2R1 Polymorphisms in the development of hepatocellular carcinoma in hepatitis C Virus-infected patients. Genet Testing Mol Biomarkers.

[23] Lange C, Miki D, Ochi H, Nischalke H, Bojunga J, Bibert S (2013). Genetic analyses reveal a role for vitamin D insufficiency in HCV-associated Hepatocellular Carcinoma Development. Plos One.

[24] Galal GM, Abudeif A, Ahmed NS (2021). Vitamin D receptor gene polymorphisms and risk of hepatocellular carcinoma in hepatitis C-related liver cirrhosis. Egypt Liver J.

[25] Neamatallah M, Serria MS, El-Bendary M (2022). Association of vitamin D gene polymorphisms with HCV infection outcome. Br J Biomed Sci.

[26] Mohammed M, Omar N, Mohammed S, Deiab A (2017). The significance of vitamin D receptor gene polymorphisms for susceptibility to hepatocellular carcinoma in subjects infected with hepatitis C virus. Gastroenterol Hepatol Open Access.

[27] Moemen Y, Khalil F, Khalil A (2019). FokI polymorphism in vitamin D receptor gene and its association with hepatocellular carcinoma in Egyptian patients with chronic liver disease. Meta Gene.

[28] Bruix J, Sherman M (2011). Management of hepatocellular carcinoma: an update. Hepatology.

[29] Yong Y, Lin H (2005). SHEsis, a powerful software platform for analyses of linkage disequilibrium, haplotype construction, and genetic association at polymorphism loci. Cell Res.

[30] Besheer T, Arafa M, El-Maksoud MA, Elalfy H, Hasson A, Zalata K (2018). Diagnosis of cirrhosis in patients with chronic hepatitis C genotype 4: role of ABCB11 genotype polymorphism and plasma bile acid levels. Turk J Gastroenterol.

[31] Wu EM, Wong LL, Hernandez BY, Ji J-F, Jia W, Kwee SA, Kalathil S (2018). Gender differences in hepatocellular cancer: disparities in nonalcoholic fatty liver disease/steatohepatitis and liver transplantation. Hepatoma Res.

[32] Nevola R, Tortorella G, Rosato V, Rinaldi L, Imbriani S, Perillo P (2023). Gender differences in the pathogenesis and risk factors of hepatocellular carcinoma. MDPI Biology.

[33] Yi SW, Choi JS, Yi JJ, Lee YH, Han KJ (2018). Risk factors for hepatocellular carcinoma by age, sex, and liver disorder status: a prospective cohort study in Korea. Cancer.

[34] Barooah P, Saikia S, Bharadwaj R, Sarmah P, Bhattacharyya M, Goswami B (2019). Role of VDR, GC, and CYP2R1 Polymorphisms in the development of hepatocellular carcinoma in hepatitis C virus-infected patients. Genet Test Mol Biomarkers.

[35] Raafat I, Eshra KA, El-Sharaby RM, Eissa R, Saied SM, Amer I, El Sharawy S (2020). Apa1 (rs7975232) SNP in the vitamin D receptor is linked to hepatocellular carcinoma in hepatitis C virus cirrhosis. Br J Biomed Sci.

[36] Du Y, Du B, Fang X, Shu M, Zhang Y, Chung H (2021). ALT flare predicts hepatocellular carcinoma among antiviral treated patients with chronic hepatitis B: a cross-country cohort study. Front Oncol.

[37] Turshudzhyan A, Wu GY (2022). Persistently rising alpha-fetoprotein in the diagnosis of hepatocellular carcinoma: a review. J Clin Transl Hepatol.

[38] Tsounis EP, Tourkochristou E, Sapsani A, Aggeletopoulou I, Lourida T, Ζisimopoulos Κ (2022). The role of vitamin D receptor polymorphisms in the course of chronic hepatitis C infection. Ann Gastroenterol.

[39] Peng Q, Yang S, Lao X, Li R, Chen Z, Wang J (2014). Association of single nucleotide polymorphisms in VDR and DBP genes with HBV-related hepatocellular carcinoma risk in a Chinese population. PLos One.

[40] Maneechay W, Boonpipattanapong T, Kanngurn S, Puttawibul P, Geater SL, Sangkhathat S (2015). Single nucleotide polymorphisms in the Gc gene for vitamin D binding protein in common cancers in Thailand. Asian Pac J Cancer Prev.

[41] Chupeerach C, Tungtrongchitr A, Phonrat B, Schweigert FJ, Tungtrongchitr R, Preutthipan S (2012). Association of Thr420Lys polymorphism in DBP gene with fat-soluble vitamins and low radial bone mineral density in postmenopausal Thai women. Biomark Med.

[42] Dai ZM, Fei YL, Zhang WG, Liu J, Cao XM, Qu QM (2015). Association of vitamin D receptor Cdx-2 polymorphism with cancer risk a meta-analysis. Medicine (Baltimore).

[43] Slatkin M (2008). Linkage disequilibrium-understanding the evolutionary past and mapping the medical future. Nat Rev Genet.

[44] Chuang SC, Lee YC, Hashibe M, Dai M, Zheng T, Boffetta P (2010). Interaction between cigarette smoking and hepatitis B and C virus infection on the risk of liver cancer: a meta-analysis. Cancer Epidemiol Biomarkers Prev.

[45] Yang TH, Chan C, Yang PJ, Huang YH, Lee MH (2023). Genetic susceptibility to hepatocellular carcinoma in patients with chronic hepatitis virus infection. Viruses.

