# Supplementary material for: Vitamin D receptor rs3782905 and vitamin D binding protein rs7041 polymorphisms are associated with hepatocellular carcinoma susceptibility in cirrhotic HCV patients
Source: BMC Med Genomics. 2023 Dec 8;16:319. doi: 10.1186/s12920-023-01749-8 (PMC10704848; doi:10.1186/s12920-023-01749-8)
Supplement: Supplementary file 1 — Additional file 1: Table 1S. Different inheritance models analysis of the SNPs between Cirrhosis and control groups. Table 2S. Different inheritance models analysis of the SNPs between HCC and control groups. [file 12920_2023_1749_MOESM1_ESM.docx]

**Table 1S**. Different inheritance models analysis of the SNPs between Cirrhosis and control groups.

| **SNPs** | **Model** | **Genotype** | **AIC** | **BIC** |
| --- | --- | --- | --- | --- |
| **VDR (rs2228570)** | **Codominant** | CC | 421.857 | 428.454 |
|  |  | CT |  |  |
|  |  | TT |  |  |
|  | **Dominant** | CC | 276.37 | 279.67 |
|  |  | CTTT |  |  |
|  | **Recessive** | CCCT | 196.489 | 199.788 |
|  |  | TT |  |  |
|  | **Over dominant** | CCTT | 265.582 | 268.881 |
|  |  | CT |  |  |
| **DBP (rs7041)** | **Codominant** | TT | 430.1 | 436.7 |
|  |  | TG |  |  |
|  |  | GG |  |  |
|  | **Dominant** | TT | 274.1 | 277.4 |
|  |  | TGGG |  |  |
|  | **Recessive** | TTTG | 212.8 | 216.1 |
|  |  | GG |  |  |
|  | **Over dominant** | TTGG | 263.4 | 266.7 |
|  |  | TG |  |  |
| **VDR (rs3782905)** | **Codominant** | GG | 442.088 | 448.684 |
|  |  | GC |  |  |
|  |  | CC |  |  |
|  | **Dominant** | GG | 260.979 | 264.277 |
|  |  | GCCC |  |  |
|  | **Recessive** | CGGG | 262.195 | 265.493 |
|  |  | CC |  |  |
|  | **Over dominant** | GGCC | 244.627 | 247.926 |
|  |  | CG |  |  |

AIC; Akaike's Information Criterion, BIC; Schwarz's Bayesian Criterion.

**Table 2S**. Different inheritance models analysis of the SNPs between HCC and control groups.

| **SNPs** | **Model** | **Genotype** | **AIC** | **BIC** |
| --- | --- | --- | --- | --- |
| **VDR (rs2228570)** | **Codominant** | CC | 422.729 | 429.316 |
|  |  | CT |  |  |
|  |  | TT |  |  |
|  | **Dominant** | CC | 270.180 | 273.473 |
|  |  | CTTT |  |  |
|  | **Recessive** | CCCT | 198.922 | 202.215 |
|  |  | TT |  |  |
|  | **Over dominant** | CCTT | 270.180 | 273.473 |
|  |  | CT |  |  |
| **DBP (rs7041)** | **Codominant** | TT | 434.9 | 441.5 |
|  |  | TG |  |  |
|  |  | GG |  |  |
|  | **Dominant** | TT | 271.0 | 274.2 |
|  |  | TGGG |  |  |
|  | **Recessive** | TTTG | 254.9 | 258.2 |
|  |  | GG |  |  |
|  | **Over dominant** | TTGG | 230.6 | 233.9 |
|  |  | TG |  |  |
| **VDR (rs3782905)** | **Codominant** | GG | 435.686 | 442.272 |
|  |  | GC |  |  |
|  |  | CC |  |  |
|  | **Dominant** | GG | 264.655 | 267.949 |
|  |  | GCCC |  |  |
|  | **Recessive** | CGGG | 228.514 | 231.808 |
|  |  | CC |  |  |
|  | **Over dominant** | GGCC | 264.655 | 267.949 |
|  |  | CG |  |  |

AIC; Akaike's Information Criterion, BIC; Schwarz's Bayesian Criterion.
